# DARE: Distill and Reinforce Ensemble Neural Networks for Climate-Domain Processing

**DOI:** 10.3390/e25040643

**Published:** 2023-04-11

**Authors:** Kun Xiang, Akihiro Fujii

**Affiliations:** Department of Science and Engineering, Hosei University, Tokyo 184-8584, Japan

**Keywords:** natural-language processing, knowledge distillation, data augmentation, domain adaptation, climate change

## Abstract

Natural-language processing is well positioned to help stakeholders study the dynamics of ambiguous Climate Change-related (CC) information. Recently, deep neural networks have achieved good results on a variety of NLP tasks depending on high-quality training data and complex and exquisite frameworks. This raises two dilemmas: (1) the networks are highly reliant on powerful hardware devices and processing is time-consuming, which is not only inconducive to execution on edge devices but also leads to resource consumption. (2) Obtaining large-scale effective annotated data is difficult and laborious, especially when it comes to a special domain such as CC. In this paper, we propose a CC-domain-adapted BERT distillation and reinforcement ensemble (DARE) model for tackling the problems above. Specifically, we propose a novel data-augmentation strategy which is a *Generator-Reinforced Selector* collaboration network for countering the dilemma of CC-related data scarcity. Extensive experimental results demonstrate that our proposed method outperforms baselines with a maximum of 26.83% on SoTA and 50.65× inference time speed-up. Furthermore, as a remedy for the lack of CC-related analysis in the NLP community, we also provide some interpretable conclusions for this global concern.

## 1. Introduction

Climate change (CC) has become a central topic within the multiple branches of social sciences. Stakeholders are widely involved in all areas from private talks to public social media, and from scientific papers to journalistic articles. Natural-language processing (NLP) is well positioned to help stakeholders study the dynamics of ambiguous CC-related information. However, we have found that the amount of work done so far on CC remains limited within the NLP community. This is in sharp contrast to the attention that CC receives in various other social disciplines.

When BERT has been proposed, pretraining the language model with a large-scale dataset and fine-tuning it with a small-scale dataset seems to be a default or even a trend because of its powerful and excellent performance on different downstream tasks. Consequently, the LM scale-up requires more computation cost, powerful GPUs, and extensive storage and burden to be applied to edge devices. On the other hand, obtaining a large-scale effective labeled dataset to train this kind of LM is always difficult. In particular, when it comes to a specific domain such as climate change, due to the scarcity of available domain data, the model performance degrades distinctly because the data distribution of the source domain and the specific domain have severely deviated.

Domain adaptation (DA) and knowledge distillation (KD) are two typical transfer-learning methods that can help resolve this dilemma. Domain adaptation is used to generally seek and identify features shared between two domains, or learn useful representations for both domains. The latter is for model compression and acceleration, which is useful for saving computational resources.

In this paper, we propose a novel CC-domain-adapted model based on knowledge distillation and reinforcement learning. The model is known as DARE, which stands for “distill and reinforce ensemble” neural networks. Specifically, to tackle the problem of CC-related annotated dataset scarcity, we propose a novel data-augmentation strategy that is inspired by computer vision (CV). In CV, unlabeled homologous images can be easily obtained by image distortion. However, when it comes to NLP, a similar noise-additive method performs badly because of ambiguous and complicated linguistics.

Therefore, we propose to randomly replace the current word with the same part of speech (POS) as a *Generator*. The *Generator* can automatically generate abundant unlabeled sentences as an augmented dataset. It is similar to the operation of synthesizing images by distortion in CV. Furthermore, considering the particularity of linguistics, we design a *Selector* based on reinforcement learning (RL) to dynamically guide and select high-quality augmented data for guiding training.

To sum up, the contributions of our work are as follows:

(1) We propose to combine knowledge distillation and domain adaptation for the processing of a large number of disordered, unstructured, and complex CC-related text data. This is a language model that combines pretraining and rule embedding, which ensures that the compression model improves training speed without sacrificing too much performance. We evaluate our approach to sentiment analysis and fact-checking NLP tasks. The results fully demonstrate the excellent performance of our proposed model, which not only surpasses the baselines on accuracy and F1 score, but also reduces the parameters of the model to complete the model acceleration.

(2) Aiming to resolve the dilemma of data scarcity within the specific domain, we propose a novel data-augmentation method which is a *Generator–Selector* collaboration network based on reinforcement learning where the *Generator* automatically generates data, and the *Reinforced Selector* guides and selects high-quality augmented data.

(3) We provide some interpretable conclusions on climate change-related text with the use of NLP tools, which provide a theoretical basis for CC-related topic followers.

The rest of this paper is organized as follows: Section 2 briefly provides a literature review of current works. Section 3 demonstrates the details of specific LM structure modules and the proposed data-augmentation method. Section 3 carries out all the experiments, and provides results and statistical explanations. Finally, we give some conclusions and discuss the deficiencies of this work. The appendixes provide details on additional experiments.

## 2. Related Work

In this section, we briefly provide a literature review of current works, mainly of three aspects that are the most relevant to the core technologies used in this work. We follow the sequence of BERT-based knowledge distillation, data augmentation and domain adaptation, and the NLP of climate change-related text.

### 2.1. Bert-Based Knowledge Distillation

As a revolutionary representation model, BERT [1] has attracted attention in natural-language processing, but it is also a cumbersome deep model that is not easy to deploy. To address this problem, several lightweight variations of BERT (called BERT model compression) using knowledge distillation have been proposed. Sun et al. [2] proposed patient knowledge distillation, which designs a student model that patiently learns from multiple intermediate layers of the teacher model for incremental knowledge extraction. Jiao et al. [3] proposed a novel transformer distillation method that was realized to compress the model into four layers. Tang et al. [4] proposed to distill knowledge from BERT into a single-layer Bi-LSTM for sentence-pair tasks. Across multiple datasets, they achieved comparable results with ELMo, while using roughly 100 times fewer parameters and 15 times less inference time. Sanh et al. [5] proposed DistilBERT to pretrain a smaller general-purpose language representation model by introducing a triple loss combining language modeling, distillation, and cosine-distance losses. Aguilar et al. [6] proposed to distill the internal representations of a large model into a simplified version to address the problem of internal representation difference.

### 2.2. Data Augmentation and Domain Adaptation

However, currently, most works on reinforcement learning or adversarial learning-based data-augmentation methods for domain adaptation are concentrated on computer vision or cross-domain knowledge transfer, and there is still a lack of work in the field of NLP. Therefore, the following literature review is not limited to the combination of these two technical means to solve the NLP problem. We have selected some representative works to illustrate.

Feng et al. [7] proposed a method to learn to augment data-scarce BERT-domain knowledge distillation, learning a cross-domain manipulation scheme that automatically augments the target with the help of resource-rich source domains to tackle the problem of performance degradation due to data scarcity in the target domain. Aiming at the same problem, Ma et al. [8] presented a novel two-step domain-adaptation framework based on curriculum learning and domain-discriminative data selection. Du et al. [9] combined adversarial learning and domain adaptivity to design a post-training procedure, which will encourage BERT to be domain-aware and distill the domain-specific features in a self-supervised way. Similarly, when conducting NLP tasks with BERT-based models, domain-adaptive strategies were introduced to improve the performance of specific downstream tasks. Such works have gradually attracted the attention of researchers [10,11,12,13,14].

### 2.3. NLP of Climate Change-Related Text

We briefly summarize works with NLP-related methodologies on CC-related data. The number of such publications is small, so we mention them here in chronological order. Henceforth, we use “CC” as the abbreviation for “climate change”.

Cody et al. [15] used an existing available measurement tool called the Hedonometer to analyze human awareness and sentiment polarities in response to CC by retrieving tweets containing the word “climate”. Similarly, Diakopoulos et al. [16] developed a novel operationalization of moral evaluation frames and study within a corpus of 3000 blogs discussing CC. Pathak et al. [17] presented a study of a large collection of Twitter data centered on the 2015 UN Climate Change Conference. They analyzed demographics, emotion, and opinion dynamics over time and location based on topic-mining models. Such similar work to mine sentiment tendency and topic analysis from Twitter or other text data is abundant [18,19,20,21,22].

Kolbel et al. [23] used BERT to quantify regulatory climate risk disclosures and differentiate between transition and physical climate risks. Luccioni et al. [24] created a custom model so-called ClimateQA based on RoBERTa, which allows the analysis of financial reports to identify climate-relevant sections based on a question-answering approach. Webersinke et al. [25] proposed ClimateBERT, which is a transformer-based language model that is further pretrained on over 1.6 million paragraphs of climate-related texts and achieved quite good results on several validation datasets.

Given the summary above, and to the best of our knowledge, we are the first to combine specific transfer-learning methods and reinforcement-learning-based data augmentation to train LM and then apply it to CC-related texts to solve problems in the real world.

## 3. Model

In this section, we present an overview of the proposed method. Specifically, the framework of our model consists of three core technologies and several modules. We will follow the workflow to introduce our proposal with the sequence of knowledge distillation, data augmentation, and domain adaptation.

### 3.1. Knowledge Distillation

The framework includes three components: the teacher model, the student model, and the distillation target. For the teacher model, we use a 12-layer BERTbase model (https://github.com/codertimo/BERT-pytorch Accessdate|7 September 2022), including a 12-layer transformer structure. The student model is a simple Bi-LSTM-Attention model. Specifically, we design a self-attention mechanism to learn to represent more accurate semantic features and incorporate part-of-speech (POS) vectors to strengthen the sentiment connection and obtain more sentiment features. The details are introduced below.

#### 3.1.1. Teacher Model

BERT [1] is composed of multiple layers of transformers, which facilitate the model to obtain long-distance dependencies between input data. Each layer of the transformer contains two main sublayers: multi-head attention (MHA) and feedforward network (FFN), which employ residual connections and layer normalization around each of the two sublayers. The output of each sublayer is *LayerNorm (x + Sublayer(x))*. To keep the connections between sublayers, all sublayers in the model, as well as the embedding layer, produce outputs of the same dimension.

*Sublayer 1*: The computation function of the multi-head attention mechanism relies on three parts: queries, keys, and values corresponding to matrices *Q*, *K*, and *V*, respectively.

(1) $$\begin{array}{cc} \mathrm{Attention}(Q,K,V) & =\mathrm{softmax}\left(\frac{QK^{T}}{\sqrt{d_{k}}}\right)V \end{array}$$

Multi-head attention refers to the result of attention computation combining multiple subspaces of different representation vectors, where *n* represents the number of subspaces and $h_{i}$ represents the vector obtained after the attention calculation in the *i*th subspace.

(2) $$\begin{array}{cc} MultiHead(Q,K,V) & =concat(h_{1},h_{2},...,h_{n})W \end{array}$$

(3) $$\begin{array}{cc} h_{i} & =Attention(QW_{i}^{Q},KW_{i}^{K},VW_{i}^{V}) \end{array}$$

*Sublayer 2*: The position feedforward network is a fully connected forward network(FFN), which is:

(4) $$\begin{array}{cc} FFN(Z) & =max\left(0,ZW_{1}+b_{1}\right)W_{2}+b_{2} \end{array}$$

#### 3.1.2. Student Model

**Figure 1:**
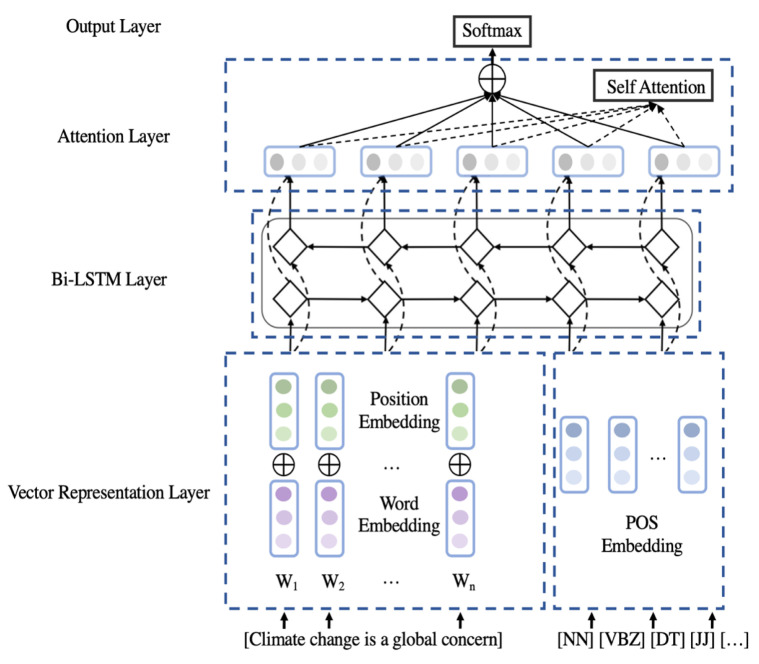
Structure of POS-Bi-LSTM-Attention.

For the student model, we use a part-of-speech-guided Bi-LSTM-Attention network, as Figure 1 shows. Long Short-Term Memory (LSTM) proposed by Hochreiter et al. [26] is a variant of RNN. Due to its design characteristics, it is often used to model contextual information in NLP tasks to better capture long-distance dependencies. Since LSTM is based on a state memory and multilayer cell structure, it can learn the information to remember and which information to forget through the training process. Bi-LSTM (Bidirectional Long Short-Term Memory) is a combination of forward and backward LSTM. In more fine-grained classification, it is necessary to pay attention to the interaction among contexts. Bi-LSTM can help better capture bidirectional semantic dependencies and help to implement backward-to-forward encoding to obtain more accurate emotional expressions. The attention mechanism has arguably become one of the most important concepts in the deep-learning field. It is inspired by the biological systems of humans that tend to focus on distinctive parts when processing large amounts of information. In a neural network, the more parameters of the model, the stronger the expression ability of the model, and the greater the amount of information stored in the model, but this will bring about the problem of information overload. Therefore, by introducing an attention mechanism, focusing on the information that is more important to the current task, and even filtering out irrelevant information. With the development of deep neural networks, the attention mechanism has been widely used in diverse application domains.

(1) Part-of-Speech Tagging

Linguistics is always ambiguous and complicated. Part of speech (POS) mainly refers to the basic attributes of words in the text, which can well represent features and is also a process of de-fuzzification. The same one word may have various parts of speech, for example, “change” in “climate change” is a noun, while it is a verb in “change the climate”. In part-of-speech tagging, to avoid this kind of ambiguity, we need to give each word in the text a unique POS tag.

(2) Structure of POS-Bi-LSTM-Attention

Currently, many neural network models have been successfully applied to sentiment classification tasks, but when it comes to climate change, which contains richer and more ambiguous semantic information, we should pay more attention to learning the essential characteristics of vocabulary. Hence, we propose a POS-Bi-LSTM-Attention model to optimize the algorithm from two points of view: the attention mechanism and part-of-speech-based word vectors. Specifically, we introduce a self-attention mechanism to learn to represent more accurate semantic features and incorporate part-of-speech vectors to strengthen the sentiment connection between words and POS to obtain more sentiment features. The model structure framework is designed as Figure 1 shows:

*Vector Representation Layer*

The vector representation layer, also the embedding layer, aims to convert the word segmentation, the position information of the word, and the corresponding POS into vectors, and feed into the model. Aiming at the polysemy, irony, ambiguity, and other specific characteristics of CC context, we use Word2Vec to generate vectors.

Assuming a segmented sentence $S=[w_{1},w_{2},w_{3},\ldots ,w_{n}]$ ($w_{i}$ represents the *i*th word in the sentence), each word in the sentence is mapped to a *d*-dimensional vector, which is word embedding. All the words in the sentence are represented by *d*-dimensional vectors, and the word-embedding matrix $S^{n*d}$ is constructed. Each word $w_{i}$ is transformed into a low-dimensional word-embedding $v_{w}^{\left(e\right)}$ using the matrix–vector product, which can be expressed as:

(5) $$\begin{array}{cc} v_{w}^{\left(e\right)} & =S^{\left(e\right)}h_{w}^{\left(e\right)} \end{array}$$

The embedding matrix $S^{\left(e\right)}\in R^{d*|v|}$, |V| is a fixed-size vocabulary, and *d* denotes the dimension of word vectors. $h_{w}$ is the one-hot encoding of $w_{i}$, and the dimension is |V|, where the value is [1] on $w_{i}$, and [0] on the others. Each column $S^{\left(e\right)}\in R^{d}$ in the embedding matrix represents the *i*th word embedding. Learned word embedding can capture semantic and structural information.

To better understand content semantics and capture important information, we propose to incorporate a self-attention mechanism into the model. Since the self-attention mechanism itself does not extract time-series features, the position-encoding vector is added along with the input word vector representation for combining the position information for achieving better results with position information. When encoding the position, although different position embedding corresponds to different positions, the association between words in different positions is inversely proportional to the distance. Considering the influence of distance on position encoding, relative position encoding is introduced. In the relative encoding process, each word can be represented as a vector $P_{i}$ with dimensions, therefore forming a position-encoding matrix $P=[P_{1},P_{2},P_{3},\ldots P_{n}]$.

Given the position index *j* of each word in a sentence, its corresponding position-encoding vector *i* can be represented as PE(i,j), and the position encoding with even and odd position indices is defined as:

(6) $$\begin{array}{c} PE\left(i,j\right)=\left\{\begin{array}{cc} sin(wk\cdot j), & \mathrm{if}\;i=2k\\ cos(wk\cdot j), & \mathrm{if}\;i=2k+1 \end{array}\right. \end{array}$$

while $w_{k}=1\backslash 1000\frac{2k}{d_{emb}}$, *k* is the dimension of position encoding, $d_{emb}$ represents the length of feature vectors. The relative positional relationship can be learned by introducing a sin and cosine transformation mechanism. The position encoding encodes the position *j* which is the given input into the sequence as a *d*-dimensional position vector. For any fixed offset *l*, $PE_{i+1}$ can be described as a linear function of $PE_{i}$, so that long-term memory can be stored. The *j*th element of the position-encoding vector $PE_{j}\left(p\right)$ can be expressed as:

(7) $$\begin{array}{cc} PE_{j} & =[sin\left(w_{l}\cdot j\right)cos\left(w_{i}\cdot j\right)\ldots sin\left(w\frac{d_{emb}}{2}\cdot j\right)cos\left(w\frac{d_{emb}}{2}\cdot j\right)] \end{array}$$

The learned embedding is used to enrich the meaning of each position of the vector *P*, and for each $P_{i}\in P$, a corresponding position embedding $v_{w}^{\left(P\right)}$ is generated, which can be expressed as:

(8) $$\begin{array}{cc} v_{w}^{\left(P\right)} & =S^{\left(P\right)}h_{w}^{\left(P\right)} \end{array}$$

where $h_{w}^{\left(P\right)}$ represents the one-hot encoding of $P_{i}$, $S^{\left(e\right)}\in R^{k*|v|}$ is the learned position embedding transformation matrix, and |V| is the number of relative position encoding, the same size as vocabulary; *k* denotes the dimension of position vectors, which is the same as word vectors. $v_{w}^{\left(P\right)}$ is the embedding vectors of *d* dimensions after conversion. The dimensions of the position vector and the word vector are consistent to facilitate the addition of vector units, and the final semantic vector representation of the word is obtained through the addition of vectors.

Part of speech provides a lot of information about a certain word and its context words, including the word’s POS category and the similarities and differences between them. Therefore, POS is introduced into the network as a feature for vector representation. After part-of-speech tagging, the POS tag of each word is generated, and each POS tag is converted into a POS feature vector by the one-hot vector, and the POS feature of word *w* is converted into POS embedding $v_{w}^{\left(pos\right)}$ through matrix–vector product, which can be expressed as:

(9) $$\begin{array}{cc} v_{w}^{\left(pos\right)} & =S^{\left(pos\right)}h_{w}^{\left(pos\right)} \end{array}$$

$W^{\left(pos\right)}\in R^{d*|U|}$ is the POS embedding matrix to be learned. |U| is the number of POS tags. $d^{\left(pos\right)}$ denotes the dimension of POS vectors, which is also the size of each column of $S^{\left(pos\right)}$. $h_{w}^{\left(P\right)}$ denotes the one-hot encoding of POS tags. $v_{w}^{\left(pos\right)}$ is the POS embedding vectors after conversion. The part-of-speech embedding vector is concatenated with the row vector of the word semantic vector, which contains position information so that the word vector will have the grammatical structure information of this word.

For each word *w*, its word-embedding $v_{w}^{\left(e\right)}$, word position embedding $v_{w}^{\left(p\right)}$ and the POS embedding $v_{w}^{\left(pos\right)}$ together constitute the input of the Bi-LSTM-Attention network.

*Self-Attention Layer*

The sentiment polarity of sentences is not only related to contextual information, but also related to sentiment words, degree adverbs, and negations. The words in the given sentence all contribute differently to the sentiment of the context. By combining $h_{t}^{\left(for\right)}$ and $h_{t}^{\left(back\right)}$, the semantics of the obtained hidden layer is denoted as $h_{t}$, and the forward and backward semantic information in the state of $h_{t}$ is equal. To capture more direct semantic dependencies and enable the model to pay attention to the important information of semantic features during the training process, we introduce an attention mechanism to solve the problem of inconsistent word sentiment contribution based on the Bi-LSTM feature extraction. The output of the intermediate state from the Bi-LSTM layer is fed into the attention mechanism, which allocates different weights to words, increasing the importance of words by allocating higher weights, therefore extracting the words with more importance to enhance the ability of sentiment understanding towards the entire text and can also improve the classification efficiency. In this paper, we also propose to adopt the self-attention layer used in the transformer, and the self-attention mechanism only pays attention to its own data.

The hidden vector $h_{t}$ generated by the Bi-LSTM neural network at each time step is used as the input of self-attention, and the Q,K,V will be obtained by multiplying the given word-embedding emb by the corresponding matrix $W_{Q}\in R^{d_{emb}*dq}$, $W_{K}\in R^{d_{emb}*dk}$ and $W_{V}\in R^{d_{emb}*dv}$. These matrices are learned by the model during the training phase, and the vector sizes dq,dk,dv are obtained by calculating $Q=embW_{Q}$, $K=embW_{K}$, and $V=embW_{V}$. *Q* is a matrix $Q=[q_{1},q_{2},\ldots q_{n}]$ containing all query vectors. $q_{i}$ is a query vector. *K* is a matrix $K=[k_{1},k_{2},\ldots k_{n}]$ containing all key vectors. $k_{i}$ is a key vector. *V* is a matrix $V=[v_{1},v_{2},\ldots v_{n}]$ containing all value vectors. $v_{i}$ is a value vector.

For self-attention, the input sources of the three matrices Q,K,V are the same. Each hidden layer representation obtained through the Bi-LSTM layer is again encoded through a self-attention mechanism for extracting higher-level feature representations. We adopted a dot product attention method in the similarity calculation in this work. The next step is to calculate the similarity between *Q* and *K* which is to calculate $XX^{T}$ in this work. The specific formula expression is:

(10) $$\begin{array}{cc} s(h_{t},h_{i}) & =h_{t}^{T}h_{i} \end{array}$$

where $h_{t}$ is the hidden state of the decoder at time *t* and $h_{i}$ is that on the *i*th position, the result of similarity calculation $s(h_{t},h_{i})$ (expressed as $e_{ti}$). Finally, we normalize with SoftMax, which can be expressed as:

(11) $$\begin{array}{c} \alpha_{t,i}=\frac{exp(e_{t,i})}{\sum_{j=1}^{N}exp\left(e_{t,j}\right)} \end{array}$$

The weight is then multiplied by the hidden vector of the corresponding word and added to the final attention value, which can be expressed as:

(12) $$\begin{array}{c} c=\sum_{i=1}^{N}\alpha_{t,i}h_{i} \end{array}$$

The entire attention value calculation process can be expressed as:

(13) $$\begin{array}{c} Attention=Softmax(\frac{XX^{T}}{\sqrt{d_{k}}})V \end{array}$$

To deal with the possible result that the dot product is too large, it is divided by an adjustment factor $\sqrt{d_{k}}$, where $d_{k}$ is the dimension of the vectors of key and value. This is to avoid the situation where the SoftMax value is either 0 or 1 due to the value of $XX^{T}$ being excessively large. The forward context representation Fc is obtained by calculating the weighted sum of the forward word vectors based on the weight α, which can be expressed as: $Fc=\sum (\alpha_{f}h_{f})$. The backward context representation Hc can be represented as: $Hc=\sum (\alpha_{b}h_{b})$. The context representation S=[Fc,Hc] is obtained by concatenating Fc and Hc. The hidden vector of each word is multiplied by its corresponding weight to obtain the vector *S*, which is regarded as the sentiment feature for sentiment polarity classification. By calculating and assigning the probability weights corresponding to different word vectors, the key information of the text is further highlighted, which is beneficial to extracting the deep features of the text.

#### 3.1.3. Distillation Goal

The goal of knowledge distillation is to transfer knowledge from a large teacher network *T* to a small student model *S*. The student model network will be trained to mimic the behavior of the teacher network. $f^{T}$ and $f^{S}$ represent the behavior functions of the teacher network and the student network, respectively. The goal of the behavior function is to transform the input of the network into a coherently encoded representation of information. Formally, knowledge distillation is modeled as the minimization process of the loss function, which is expressed as:

(14) $$\begin{array}{c} L_{KD}=\sum_{x\in D}L\left(f^{S}\left(x\right),f^{T}\left(x\right)\right) \end{array}$$

where L(·) is the loss function that measures the gap between the teacher network and the student network, *x* is the input of the sample, and *D* is the sample dataset. Therefore, the essence of knowledge distillation is to define an effective loss function and minimize it. In BERT-based knowledge distillation, the total distillation target is composed of the word-embedding layer output, the encoding layer, and the prediction layer.

(1) Output prediction layer distillation
  (15) $$\begin{array}{cc} y_{i}^{T} & =softmax\left(z^{T}\right)=\frac{exp(z_{i}^{T})}{\sum_{j}exp\left(z_{i}^{T}\right)} \end{array}$$
  (16) $$\begin{array}{cc} L_{pre} & =MSE(y^{T},y^{S}) \end{array}$$
  where $y^{T}$ is the status of the output of the teacher model.
(2) Hidden layer distillation

There are many intermediate variables in the encoding layer of the transformer. In the BERT model, the pretrained attention distribution weights can capture rich linguistic knowledge, including the co-occurrence relationship between grammar and words. Therefore, with the help of attention-based distillation learning, it helps the teacher model to transfer its learned linguistic knowledge to the student model. Students can acquire relevant knowledge by learning the teacher’s multi-head attention matrix, which can be formulated as:

(17) $$\begin{array}{c} L_{attn}=\frac{1}{h}\sum_{i=1}^{h}MSE\left(A_{i}^{T},A_{i}^{S}\right) \end{array}$$

where *h* is the number of attention heads, and $A_{i}$ is the attention distribution matrix corresponding to the *i*th attention head of the teacher model. The minimum MSE is set as the loss function. In the calculation of the attention-related distillation loss function, the attention matrix is not normalized, which means the calculated $A_{i}$ is obtained without SoftMax.

(18) $$\begin{array}{cc} L_{hidn} & =MSE(H^{T},H^{S}W_{h}) \end{array}$$

$H^{T}$,$H^{S}$ denotes the hidden state corresponding to the teacher model and student model, respectively. Since the dimension of the hidden state of the student model is inconsistent with the teacher model, the weight matrix *W* is added to perform a linear transformation on the hidden state of the student, to ensure that they can calculate the minimum MSE loss function in the same dimensional space.

(3) Embedding layer distillation

(19) $$\begin{array}{cc} L_{emb} & =MSE(E^{T},E^{S}W_{e}) \end{array}$$

$E^{T}$,$E^{S}$ denotes the embedding layer corresponding to the teacher model and student model, respectively. The weight matrix *W* plays the same role as above.

The total distillation target $L_{model}$ which is also the cross-entropy loss between the soft targets of the teacher model and the student model:

(20) $$\begin{array}{cc} L_{model} & =\left\{\begin{array}{cc} L_{pred}\left(S,T\right), & layer=0\\ L_{hidn}\left(S,T\right)+L_{att}\left(S,T\right), & Max\geq layer>0\\ L_{emb}\left(S,T\right), & Max=layer+1 \end{array}\right. \end{array}$$

Max denotes the max layer numbers.

Finally, the total distillation loss of the student model can be formulated as:

(21) $$\begin{array}{cc} L & =\alpha L_{CE}+\left(1-\alpha \right)L_{model} \end{array}$$

Thus, the objective of KD is defined as:

(22) $$\begin{array}{cc} L_{KD} & =\sum_{x\in X}L\left(f^{S}\left(x\right),f^{T}\left(x\right)\right) \end{array}$$

where $f^{S}$ and $f^{T}$ represent the features of the student model and teacher model, respectively. $L_{KD}\left(\cdot \right)$ is the loss function evaluating the gap between the two models.

### 3.2. Data Augmentation

In this section, we will introduce our proposed reinforcement-learning-based data-augmentation method, which is a collaboration of *Generator* and *Reinforced Selector*. In the field of computer vision, there are affluent homologous images. Usually, augmented data can be generated by synthesizing images through distortion methods such as adding noise [27]. Inspired by this, we propose a POS-guided word-replacement method, which randomly replaces the current word with the same POS word as a *Generator*. To preserve the original training distribution, the new word is sampled from the unigram word distribution and re-normalized by the POS tag. For example, *“climate change is causing Himalayan glaciers to melt”*, *“climate change is causing Himalayan volcanoes to melt”*, and *“climate change is causing Himalayan glaciers to freeze”* are totally different semantics.

**Figure 2:**
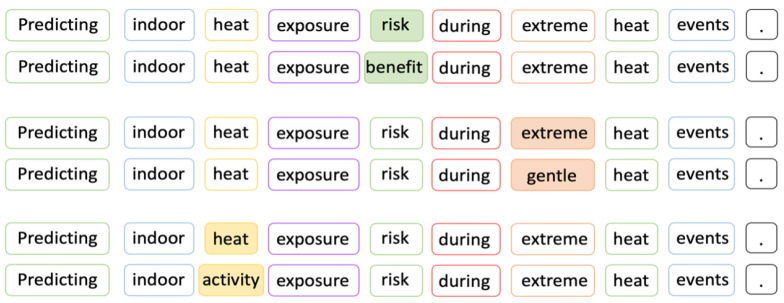
Examples of POS Tag-based Word Substitution.

First, we replace nouns, verbs, adjectives, and adverbs (denoted as $POS_{NN}$, $POS_{VB}$, $POS_{JJ}$, $POS_{RB}$, respectively), that have a greater impact on sentence semantics as Figure 2 shows. Then, with respect to the embeddings of tagged words and the words in a thesaurus, we can obtain a bunch of matched words. For example, we want to replace the word *“climate”*, which the word sequence is $\left[R=climate,w_{1},w_{2},\ldots w_{i},\ldots w_{n}\right]$, and we can obtain a tagged sequence $\left[R=wpos_{NN},w_{1},w_{2},\ldots w_{i},\ldots w_{n}\right]$ after labeling. $w_{i}$ denotes the word chosen to be the substitute. Finally, we build the newly generated data D’ by replacing the selected words.

However, as the example shows, POS replacement is easy to apply while leading to ambiguity problems and semantic-affected [28]. In contrast to adding noise to the image, making it possible to obtain abundant homologous imagines, in NLP, POS replacement may cause the student model to not effectively learn useful information representation due to the completely changed semantics.

**Figure 3:**
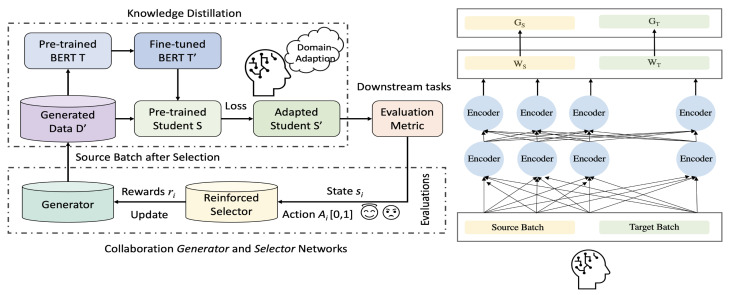
Integrated Structure of Distill and Reinforce Ensemble Neural Networks (DARE).

Thus, we design a *Reinforced Selector* based on reinforcement learning (RL) to dynamically select and guide high-quality augmented data. The goal of the *Selector* is to automatically select high-quality samples from the augmented data obtained from the *Generator*. The selection criteria are based on assessing whether the chosen sample can improve performance on validation and automatically updating it according to the *reward*. The specific collaboration structure is illustrated in Figure 3.

#### 3.2.1. State

The state vector of sample $x_{i}$ mainly consists of two components: the teacher model output $f^{T}\left(x_{i}\right)$ and the student model output $f^{S}\left(x_{i}\right)$. The concatenation is considered to be the final state vector $s_{i}^{\left(k\right)}$.

#### 3.2.2. Action

The action value of the *reinforced selector* for every sample $x_{i}$ is 1 or 0. A value of 1 represents the action to retain the sample while 0 represents the action to remove it. After obtaining the action, the student model is updated with:

(23) $$\begin{array}{cc} L_{KD} & =\sum_{x\in X}L\left(f^{S}\left(x\right),f^{T}\left(x\right)\right) \end{array}$$

#### 3.2.3. Reward

Since the criteria of the selection are based on the assessment of performance on validation, the reward $r_{i}$ is obtained by:

(24) $$\begin{array}{cc} r_{i} & =L\left(y_{i},f^{S}\left(x_{i}\right)\right)-L^{\prime }\left(y_{i},f^{S}\left(x_{i}\right)\right) \end{array}$$

where L(·) denotes the performance after updating while $L^{\prime }(\cdot )$ denotes the previous one. L(·) is set to be different according to specific tasks theoretically. However, in this work, the downstream tasks all belong to classification tasks, so L(·) is set to be the accuracy of the validation data.

## 4. Experiments

In this section, we report the dataset used in the experiments, the detailed implementation parameter setup, and a series of experimental results and statistical analysis on downstream tasks.

### 4.1. Dataset

The specific dataset used for domain-adaptive pretraining and sentiment analysis downstream task is from Berrang et al. [29], as Table 1 shows. The dataset includes the scientific literature on climate change and health published between 1 January 2013 and 9 April 2020, which is indexed in English. The search scope covers Web of Science Core Collection and Scopus. After data augmentation, the total unique record of the domain pretraining dataset is 80,750. For evaluation, we split the augmented dataset into 75% training data and 25% validation data.

For our sentiment analysis experiment, we use the dataset consisting of 1220 hand-selected paragraphs from Web of Science records before augmenting and 1000 paragraphs from Scopus records before augmenting for verification. All paragraphs were annotated as negative (risk), positive (opportunity), or neutral. The software used for collecting annotations was Prodigy (https://prodi.gy Accessdate|21 July 2022). The detailed annotation rules are explained in Appendix C.

### 4.2. Implement Details

We report implementation details below, including parameter settings, downstream tasks with statistical descriptions, and baselines for comparison.

#### 4.2.1. Parameter Settings

We followed the BERTbase (https://github.com/codertimo/BERT-pytorch Accessdate|7 September 2022) settings as the teacher model with the hidden vector of each layer as 768 dimensions, and the forward propagation vector is 3072 dimensions. We initialized our student model POS-Bi-LSTM-Attention with either 150 or 300 hidden units and 200 or 400 units in the activated hidden layer according to different validation datasets. We followed the traditional 300-dimensional Word2Vec embeddings. All the models were conducted on a single Nvidia 16 GB V100 GPU. For the KD part, we tuned the temperature to α = 0.5 and T = 1. The batch size was chosen from 8, 16, 32 and the initial lr was set to be 0.001 for the transfer-learning module and 0.02 for the policy network. The hidden layer of the policy network was set to 128 (refer to Appendix A for details). We used the Adam optimizer for the reinforced selector, with $\beta_{1}$ = 0.9, and $\beta_{2}$ = 0.999, respectively.

#### 4.2.2. Downstream Tasks

We chose to verify the model performance on the following downstream tasks: sentiment analysis and fact-checking, and the details are shown in Table 2.

#### 4.2.3. Baselines

We would like to highlight that, to better demonstrate the effectiveness of POS tagging in the word-embedding phase and domain knowledge in the pretraining phase, we also set two groups of comparative experiments, as Table 3 shows.

### 4.3. Experimental Results

We report all the conducted experiments and statistical explanations in this section.

#### 4.3.1. Sentiment Analysis

As the results show in Table 4, the two groups of comparative experiments demonstrate the effectiveness of domain pretraining and POS tagging in a word embedding. Domain pretraining improves the model with 2.64% accuracy and 2.58% F1 score, respectively. The POS tagging improves the model with 24.13% accuracy and 22.60% F1 score, respectively. This accounts for domain pretraining, which enables the model to be much more adaptive to specific downstream tasks. Compared to training BERTbase with the original large-scale open-resource dataset, small-scale domain dataset pretraining outperforms the same domain-specific downstream tasks. In addition, the model can have a better ability to obtain contextual information with POS tagging, which can alleviate semantic ambiguity to a certain extent.

Furthermore, for the sentiment analysis downstream task, our model retains 93.35% and 96.03% performance of the BERTbase (the teacher model) on accuracy and F1 score, respectively. Compared with BERTbase and DistilBERT, our model is slightly inferior in performance due to its lighter structure, but can almost have comparable performance, its accuracy is higher than other baselines by 0.34% to 26.83%, and its F1 score is higher than other baselines by 2.17% to 21.14%.

We would like to highlight that we also carried out a topic-mining experiment after obtaining sentiment polarities. Please refer to Appendix B for more details.

#### 4.3.2. Fact-Checking

To validate our model on the fact-checking downstream task, we used the dataset CLIMATE-FEVER proposed by Diggelmann et al. [31]. This dataset comprises 1.5 k sentences that make claims about climate-related topics. The authors found that the subtle complexity of modeling real-world climate-related claims within the fever framework provided a valuable challenge for general natural-language understanding.

The claims, together with their top five evidence sentences as retrieved, are displayed to the annotators to label them as supporting, refuting, or not giving enough information to validate the claim. The claim label is by default NOT_ ENOUGH_ INFO unless there is supporting (SUPPORTS) or refuting (REFUTES) evidence. If there is both supporting and refuting evidence, the claim label is DISPUTED. The details are described in Appendix C.

Here, we follow the baselines settings as per Diggelmann et al. [31] and Webersinke et al. [25] for fairness.

As shown in Table 5, for the fact-checking downstream task, our model retains 98.77% and 98.27% performance of the BERTbase (the teacher model) on precision and Macro F1, respectively. It outperforms other baselines by 0.63% to 5.25% on precision, and outperforms other baselines by 0.312% to 10.14% on Macro F1.

#### 4.3.3. Additional Exploring Experiments

However, the inferior results from the lightweight model, which has a simpler network structure, and the risk of losing knowledge is more or less the same as during the distilling of knowledge from the teacher model to the student model. This goes hand in hand with lightweight models, which means a faster training speed and consuming fewer computational resources. Hence, we carried out a sped-up experiment.

As Table 6 shows, our model has approximately good performance as BERTbase and DistilBERT, but with 50.65× and 12.66× speed-up of inference, respectively. For fairness, all the experiments were conducted on a single 16 GB V100 GPU. We performed model inference with a batch size of 512, our approach used 11.53× and 2.88× fewer parameters than BERTbase and DistilBERT, respectively, which indicates the effective breakthrough of our approach. Our model had the superiority of model acceleration without sacrificing too much accuracy.

**Figure 4:**
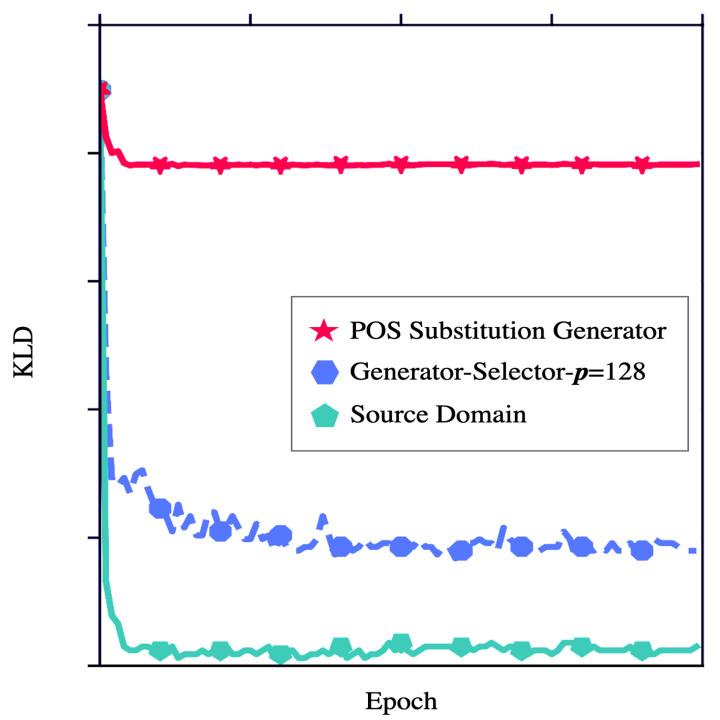
KL divergence empirical curve of reinforcement learning-based *Generator–Selector* collaboration networks.

To assess the effectiveness of the *Generator–Selector* collaboration mechanism, we conducted an experiment based on Kullback–Leibler divergence (KLD), as Figure 4 shows. KL divergence, also known as relative entropy (RE), can measure the gap of distribution between two probabilities.

By treating the normalized values of the dataset as discrete probability distributions, we can calculate the KL divergence to validate the effectiveness of the *selector* mechanism. In this case, considering the dataset before and after the reinforced *selector*, the KL divergence measures the difference between the source and the generated datasets.

The experiments above are all carried out based on optimizer parameters $\beta_{1}$ = 0.9, $\beta_{2}$ = 0.999. Figure 4 indicates that only using POS substitution to generate data can lead to considerable noise, which can cause a significant offset from the source data. This may also lead to the risk of gradient explosion and fitting problems. Therefore, it is important to design the data generation process to ensure that the generated data are of high quality and are similar to the source data.

The introduction of the reinforcement learning-based *selector* can help alleviate this plight to a certain extent. By automatically selecting high-quality data and continuously updating iteration, the *selector* can improve the quality of the generated data and improve the overall performance of the model.

To further explore the necessities of different phases of critical technologies, we conducted an ablation experiment, as Table 7 shows. We ablate the phase of knowledge distillation, the phase of domain pretraining, and the phase of data augmentation stage by stage. The results indicate the performance of the model is declining with the ablation. It demonstrates that in the case that the source domain and target domain data are offset, the model is more adaptable for specific downstream tasks by introducing domain knowledge. In addition, knowledge distillation can make the student model with a simpler structure mimic the teacher model with a more complex structure, so that knowledge can be transferred as much as possible, which cannot only help model acceleration but also enables the generated model to have domain adaptability and overcome the catastrophic forgetting problem because of the scarcity of an effectively labeled dataset.

## 5. Conclusions

Recently, people have been calling for attention to be paid to the impact on the climate and environment of deep neural networks, since their use has rocketed accompanied by energy consumption and carbon emissions required for training deep models that go far beyond people’s imagination. There is staggering consumption involved in the execution of training large-scale data on high-computing-power devices. This also causes us to reflect on our attitude toward deep learning: the optimal situation should break through the burden of edge devices and laborious training datasets.

However, even if many examples are sampled from a source, a domain gap exists because of the many linguistic variants, especially when it comes to a dataset such as CC that is so ambiguous and difficult to comprehend. In this work, we proposed a novel CC-domain-adapted model called DARE based on knowledge distillation and reinforcement learning for tackling the prevalent problems in NLP. Specifically, we proposed a novel data-augmentation strategy for countering the dilemma of CC-related data scarcity, which is implemented with a *Generator* and *Reinforced Selector* collaboration neural network.

Extensive experimental results demonstrate that our proposed method outperforms baselines with a maximum of 26.83% on SoTA and 50.65× inference time speed-up. Furthermore, as a remedy for the lack of CC-related analysis in the NLP community, we also provide some interpretable conclusions for this global concern. Extension experiments are demonstrated in the appendixes.

However, our work still leaves spaces that are worth investigating. For example, we replaced only one POS tag at a time in this work, but substituting two or even three POS tags at a time can generate exponentially more unlabeled datasets for us. Consequently, this gives our model room for improvement in the future.

Moreover, we decided not to include a wide coverage of CC-related data such as Twitter, because we assume that these texts are too noisy. However, it is well known that Twitter is quite a useful social platform for integrating views from people all around the world. Such kinds of views are more likely to represent comprehensive perspectives from different stakeholders. Hence, in the future, we may consider using this kind of data, and will also further explore advanced applications of this method and the possibility of optimizing the algorithm.

## Figures and Tables

**Table 1:** Dataset statistics before and after augmentation. “*train*” represents the training dataset, and “*dev*” is the validation dataset. The splitting ratio is 75% and 25%. “w/o DA” denotes without data augmentation and “w/” denotes with data augmentation.

Source	Records w/o DA	Records w/DA
**Web of Science**	21,734	43,292
**Scopus**	18,937	37,458
**Total Records**	40,671	80,750 (60,560 train/20,190 dev)

**Table 2:** Statistics of the dataset used in different downstream tasks.

Downstream Tasks	Dataset Usage	Labels	Label Distribution
Sentiment Analysis	Hand-selected Domain-specific ${}^{1}$	Opportunity/Neutral/Risk	872/900/448
Fact-checking	CLIMATE-FEVER ${}^{2}$	Claim:Support/Refute	1943/802

^1^ The details are described in Appendix C. ^2^ https://www.sustainablefinance.uzh.ch/en/research/climate-fever.html Accessdate|11 October 2022; Examples are sampled in Appendix C.1.

**Table 3:** Baselines and loss function and external dataset usage.

Model	Loss Functions	External Data Usage
**BERTbase**	$CE_{MLM}$+$CE_{NSP}$	✘
**BERTbase** (Domain pretrained)	$CE_{MLM}$+$CE_{NSP}$	✔(domain)
**TinyBERT** [3]	$MSE_{attn}$ + $MSE_{hidn}$ + $MSE_{emb}$ + $CE_{KD}$	✔(unlabeled + labeled)
**DistilBERT** [5]	$CE_{KD}$ + $Cos_{KD}$ + $CE_{MLM}$	✔(unlabeled)
**ClimateBERT** [25]	$CE_{MLM}$+$CE_{KD}$	✔(unlabeled + labeled)
**CCLA + Max-Pooling** [30]	CE	✔(unlabeled)
**Bi-LSTM-Attention**	CE	✘
**POS-Bi-LSTM-Attention**	CE	✘
**DARE** (ours)	$MSE_{attn}$ + $MSE_{hidn}$ + $MSE_{emb}$ + $CE_{KD}$	✔(unlabeled + labeled + domain)

**Table 4:** Performance of sentiment analysis on the domain-specific dataset. ◊ and ♣, ♡, and ♠ denote two groups of comparative experiments, which are related to the effectiveness of domain pretraining and the effectiveness of POS tagging in the word-embedding phase, respectively. The underlines denote comparatively better performance.

Model	Acc.	F1
BERTbase	◊0.947	◊0.931
BERTbase (Domain pretrained)	♣0.972	♣0.955
TinyBERT	0.871	0.870
DistilBERT	0.914	0.899
ClimateBERT	0.884	0.875
CCLA+Max-Pooling	0.824	0.829
Bi-LSTM-Attention	♡0.721	♡0.719
POS-Bi-LSTM-Attention	♠0.895	♠0.882
DARE(ours)	**0.903**	**0.894**

**Table 5:** Performance evaluation on fact-checking downstream task. “w/o NOT EI” denotes without Not Enough Information to be fair with the baselines by filtering out the NOT_ ENOUGH_ INFO as the original experimental settings. † denotes the best performance and ‡ denotes the second best one.

Model	Pre.	Macro F1	Pre. w/o NOT EI	Macro F1. w/o NOT EI
BERTbase	†0.812	†0.807	†0.801	†0.791
RoBERTa	0.782	0.723	0.735	0.712
DistilRoBERTa	0.762	0.720	0.724	0.704
ClimateBERT	0.773	0.768	0.749	0.729
DARE(ours)	‡0.802	‡0.793	‡0.793	‡0.788

**Table 6:** Experimental results of the speed-up. All experiments are conducted on a single NVIDIA 16 GB V100 GPU. “Of param.” denotes the number of millions of parameters, and the inference time is in seconds.

Model	Of Param. (M)	Inference Time (s)	Speed-Up (Times)
BERTbase	83.7/11.53×	973.41	50.65×
DistilBERT	20.9/2.88×	243.35	12.66×
DARE (ours)	7.26/1.00×	19.22	1.00×

**Table 7:** Ablation experiments of different phases of critical technologies. “w/o” denotes without, and † denotes the best performance.

Model	Acc.	F1
Basic DARE	†0.903	†0.894
w/o Knowledge Distillation	0.887	0.871
w/o Domain Pretraining	0.862	0.858
w/o Data Augmentation	0.845	0.833

**Figure A1:**
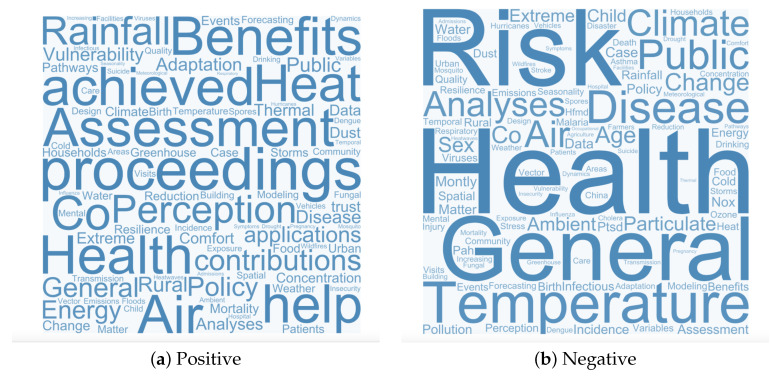
Topic mining word cloud for positive and negative sentiment polarities.

## Data Availability

Lea Berrang-Ford and et al. 2021. Systematic mapping of global research on climate and health using machine learning; DOI: https://doi.org/10.5281/zenodo.4972515 Access date|17 July 2022.

## Appendix A

**Table A1:** How different POS tags replacement act on experiment results and the relationship between different sizes of the hidden layer of the policy network. † denotes the optimal performance while the underline denotes the second-best one.

POS Replacement	32	64	128	256	512
** $POS_{NN}$ **	81.45	82.78	85.33	84.21	83.10
** $POS_{VB}$ **	85.74	86.14	86.77	85.39	84.32
** $POS_{JJ}$ **	84.65	86.34	88.94	85.76	85.23
** $POS_{RB}$ **	88.56	87.43	†89.27	86.32	85.88

We investigate how different POS tags replacement act on experiment results and the relationship between different sizes of the hidden layer of the policy network.

The results show that the optimal size of the hidden layer of the policy network is set to be 128, so the following experiments are all carried out with the hidden layer as 128. Apparently, adverb replacement affects more of the semantics and are followed by adjectives. We may also try to replace two or three kinds of POS tags at the same time in the future.

## Appendix B

In the sentiment analysis section, we also conducted an experiment with the combination of TF-IDF and LDA topic models to mine opinions for both positive and negative sentiment polarities as Figure A1 shows. The topics of positive polarity generally focus on the reports or researches that fill the gaps of what is currently lacking. Or some stakeholders are taking explicit actions dealing with the status quo. The negative polarity always discloses risks of climate change. However, we found that the results did not show a definite topic difference between the two sentiment polarities, that is, the attitudes of the two groups didn’t diverge in a way. This is an interesting discovery, arguably, that climate change-related texts are so ambivalent and ambiguous that exist in the form of conflicting information. That makes sense because risks come with opportunities to a certain extent for climate change-related stakeholders.

## Appendix C

**Label**	**Example**
Opportunity	We use a case study of the Australian island-state of Tasmania to demonstrate the importance and particularity of place in the formation of climate change adaptation issues, problem definition and framing, and the dynamics of knowledge and praxis development across a range of research and industry sectors. We describe the significance of the place Tasmania with regard to its geographical location; its portrayal as an island place; and its cultural meaning and relations. Through a synthesis of climate change adaptation research, policy literature and engagement with researchers and stakeholders, we identify three emergent thematic place characterisations of Tasmania. We find that these characterisations have contributed directly or indirectly to the: initiation and extent of research and practical activities; the framing of adaptation issues and perspectives on potential adaptation responses in different sectors including the marine biodiversity and resources sector, small business and human health sectors. Exposing these influences is essential for focusing future adaptation activities, including research, planning, investment and practice, in Tasmania and other locations where place is a central issue.
Opportunity	Adoption and adaptation of the approach used may be valuable for public health organizations to assist their communities. Through completing a vulnerability assessment, an evidentiary base was generated for public health to inform adaptation actions to reduce negative health impacts and increase resiliency. Challenges in completing vulnerability assessments at the local level include the framing and scoping of health impacts and associated indicators, as well as access to internal expertise surrounding the analysis of data. While access to quantitative data may be limiting at the local level, qualitative data can enhance knowledge of local impacts, while also supporting the creation of key partnerships with community stakeholders which can ensure climate action continues beyond the scope of the vulnerability assessment.
Opportunity	Coordinated adaptation efforts can reduce heat’s adverse health impacts, however. To address this concern in Ahmedabad (Gujarat, India), a coalition has been formed to develop an evidence-based heat preparedness plan and early warning system. This paper describes the group and initial steps in the plan’s development and implementation. Evidence accumulation included extensive literature review, analysis of local temperature and mortality data, surveys with heat-vulnerable populations, focus groups with health care professionals, and expert consultation. The findings and recommendations were encapsulated in policy briefs for key government agencies, health care professionals, outdoor workers, and slum communities, and synthesized in the heat preparedness plan. A 7-day probabilistic weather forecast was also developed and is used to trigger the plan in advance of dangerous heat waves. The pilot plan was implemented in 2013, and public outreach was done through training workshops, hoardings/billboards, pamphlets, and print advertisements. Evaluation activities and continuous improvement efforts are ongoing, along with plans to explore the program’s scalability to other Indian cities, as Ahmedabad is the first South Asian city to address heat-health threats comprehensively.
Risk	The article concludes that climate change reduces access to drinking water, negatively affects the health of people and poses a serious threat to food security.
Risk	Dengue is a major international public health concern, one of the most important arthropod-borne diseases. More than 3.5 billion people are at risk of dengue infection and there are an estimated 390 million dengue infections annually. This prolific increase has been connected to societal changes such as population growth and increasing urbanization generating intense agglomeration leading to proliferation of synanthropic mosquito species.
Risk	Agriculture in Africa is not only exposed to climate change impacts but is also a source of greenhouse gases (GHGs). While GHG emissions in Africa are relatively minimal in global dimensions, agriculture in the continent constitutes a major source of GHG emissions. In Ghana, agricultural emissions are accelerating, mainly due to ensuing deforestation of which smallholder cocoa farming is largely associated. The sector is also be devilled by soil degradation, pests, diseases and poor yields coupled with poor agronomic practices.
Neutral	This is a preliminary investigation on the environmental quality of the city of Pujili, made from the collection of samples of particulate matter and vehicular traffic counts on six points of the city. The methodology is based on the provisions of the Unified Text of Secondary Environmental Legislation for measuring atmospheric particulate matter, and the use of count tables for vehicle registration. The results reflect the impact of vehicular traffic, the characteristics of the rolling road layer, soil erosion, and climate on air pollution and its impact on the health of the population.
Neutral	The aim of the present study is to analyse the age effect on the lag patterns of relative risk of hospitalization for acute myocardial infarction and NO2, PM10 and O-3. Daily hospitalizations for AMI during the period 2008–2011 were extracted from administrative data. Analyses were performed using the quasi-Poisson regression model adjusted for seasonality, long-term trend, day of the week and temperature. We observed very different patterns depending on age. For NO2 and PM10, the younger group (25–54years) shows a more delayed effect in comparison with the two older age groups (55–64 and >=65 years). Overall, the associations between NO2 and AMI are higher compared to PM10. There are no associations between O-3 and AMI. This study indicates that age plays a major role in the lag pattern. Younger people have delayed effects, but they are nevertheless sensitive to air pollution.

In the sentiment analysis experiment, we use hand-selected paragraphs for training and verifying. We followed the rules of annotation as follows. The annotators were asked to annotate the paragraphs to be a risk(negative) or opportunity(positive). The paragraph can also make just a Neutral statement.

To be more precise, we define a paragraph as a risk(negative) if the paragraph talks about climate change affecting public health; climate change leading to serious consequences, or the trend is deteriorating. When the paragraph is economic and business related, greenwashing is a specific concept relating to negative sentiment. We define a paragraph relating to an opportunity(positive) if the paragraph is talking about climate change bringing potential opportunities to some stakeholder; an entity is taking positive actions to the situation; the research provides evidence or fills the gap of what is currently lacking. Besides, the paragraph is defined to be neutral if it only states facts or statistics without any perspective or standing for any stakeholder.

For some examples, please refer to the Appendix C.1.

### Appendix C.1

Examples taken from CLIMATEFEVER.

**Claim**: More than 100 percent of the warming over the past century is due to human actions
**Claim Verdict Label**: SUPPORTS
**Evidence #1** The view that human activities are likely responsible for most of the observed increase in global mean temperature (“global warming”) since the mid-20th century is an accurate reflection of current scientific thinking. [wiki/Kyoto_Protocol]
**Evidence #2** The dominant cause of the warming since the 1950s is human activities. [wiki/Scientific_consensus_on_climate_change]
**Claim**: Extreme weather isn’t caused by global warming
**Claim Verdict Label**: REFUTES
**Evidence #1** Researchers have for the first time attributed recent floods, droughts and heat waves, to human induced climate change. [wiki/Extreme_weather]
**Evidence #2** The effects of global warming include rising sea levels, regional changes in precipitation, more frequent extreme weather events such as heat waves, and expansion of deserts. [wiki/Global_warming]
